# *ITPR1* gene p.Val1553Met mutation in Russian family with mild Spinocerebellar ataxia

**DOI:** 10.1186/s40673-016-0040-8

**Published:** 2016-01-13

**Authors:** M. I. Shadrina, M. V. Shulskaya, S. A. Klyushnikov, T. Nikopensius, M. Nelis, P. A. Kivistik, A. A. Komar, S. A. Limborska, S. N. Illarioshkin, P. A. Slominsky

**Affiliations:** Institute of Molecular Genetics RAS, Moscow, Russia; Department of Neurogenetics, Research Center of Neurology, Russian Academy of Medical Sciences, Moscow, Russia; Estonian Genome Centre, University of Tartu, Tartu, Estonia; Center for Gene Regulation in Health and Disease, Department of Biological, Geological and Environmental Sciences, Cleveland State University, Cleveland, OH 44115 USA

**Keywords:** Spinocerebellar ataxia, *ITPR1*, Whole-exome sequencing, Sanger sequencing

## Abstract

**Background:**

Spinocerebellar ataxias (SСAs) are a highly heterogeneous group of inherited neurological disorders. The symptoms of ataxia vary in individual patients and even within the same SCA subtype. A study of a four-generation family with autosomal dominant (AD) non-progressive SCA with mild symptoms was conducted. The genotyping of this family revealed no frequent pathogenic mutations. So the objective of this study was to identify the genetic causes of the disease in this family with the technology of whole-exome sequencing (WES).

**Methods and results:**

WES, candidate variant analysis with further Sanger sequencing, mRNA secondary structure prediction, and RSCU analysis were performed; a heterozygous missense mutation in *ITPR1* was identified.

**Conclusion:**

Our study confirms the fact that *ITPR1* gene plays a certain role in the pathogenesis of SCAs, and, therefore, we suggest that c.4657G>A p.Val1553Met) is a disease-causing mutation in the family studied.

## Background

Spinocerebellar ataxias (SСAs) are a highly heterogeneous group of inherited neurological disorders. They are characterized by significant clinical polymorphism resulting from combination of cerebellar ataxia with additional non-cerebellar symptoms, which seriously complicates clinical differentiation [1]. The symptoms of ataxia vary in individual patients and even within the same SCA subtype. In general, affected individuals exhibit progressive cerebellar ataxia variably associated with dysarthria, oculomotor abnormalities, pyramidal and extrapyramidal signs, epilepsy, and mental impairment. Neuronal loss is observed predominantly in the cerebellum and the brainstem, and neuroimaging (CT or MRI) demonstrates the atrophy of those regions. These pathologies can be caused by autosomal dominant (AD), autosomal recessive, and X-linked mutations.

There are many different SCA classifications based on both clinical and anatomical principles. Harding suggested a classification according to the mode of inheritance and clinical signs [2]. The actual numbering corresponds to the order of gene description. This molecular classification is currently the most accepted by the scientific community. To date, 38 types of AD SCAs have been identified [3], which are difficult to distinguish clinically. There are several common forms of the disease in each geographical region (such as SCA1, SCA2, SCA3 and some others in Russia) with dynamic mutations resulting in most severe clinical manifestations, and these forms are usually diagnosed in clinical practice [4]. Other forms of AD SCAs are rare, occur predominantly in a small number of families, exhibit relatively mild manifestations, and are difficult to define because of some phenotypic overlap between them.

Here, a study of a four-generation family with AD non-progressive SCA with mild symptoms was conducted. The genotyping of this family revealed an absence of pathogenic mutations that are common for the Russian population. In such cases for which the screening of known mutations is negative, whole-exome sequencing (WES) can be used in order to find new or rare mutations in the coding regions of the genome. We applied this technology in our family with AD SCA.

## Methods

### Human subjects

**Fig. 1:**
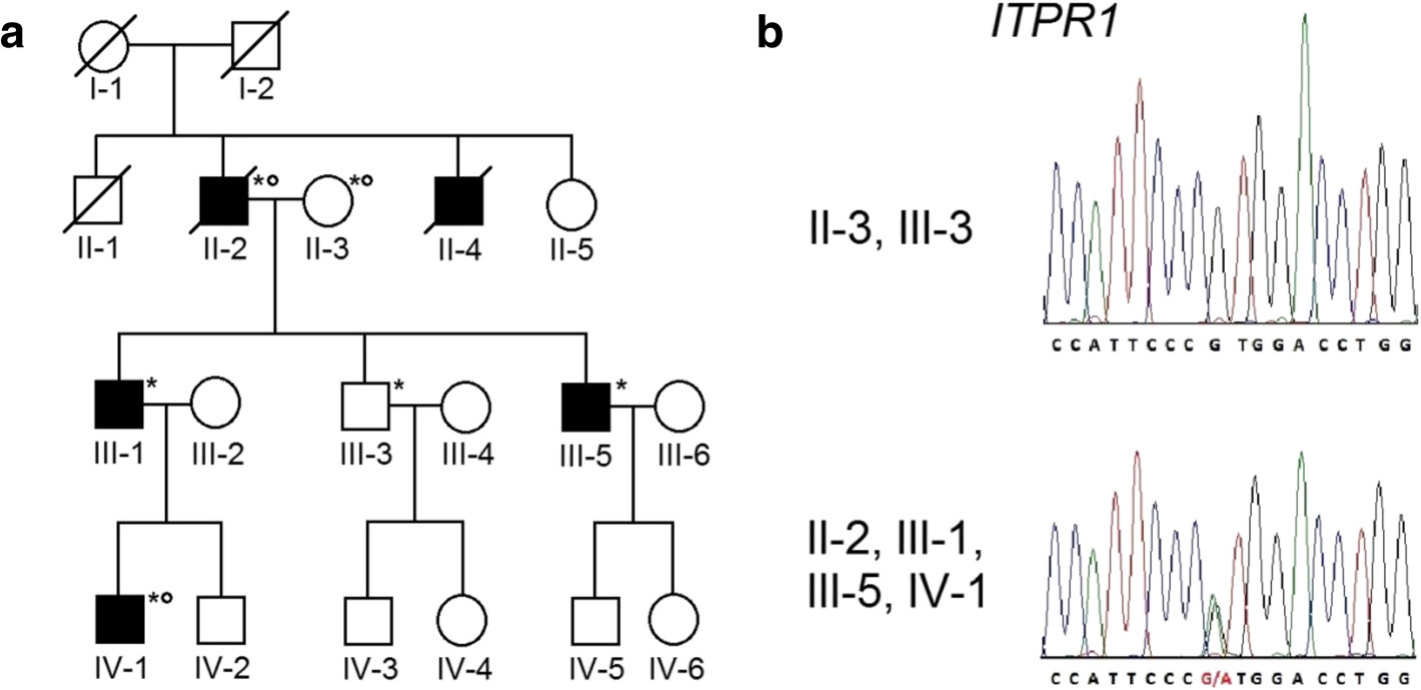
Pedigree and sequence chromatograms of the family. **a** - Small circles on the top right indicate members for whom whole-exome sequencing was carried out. *Asterisks* indicate members for whom the PCR and Sanger sequencing validation were carried out. **b** - Red letters show the heterozygous substitution c.4657G>A in the affected individual

Informed consent was obtained from all participating patients and families according to the Declaration of Helsinki, and the studies were approved by the Local Ethics Committee of the Research Center of Neurology, Moscow. The pedigree of this family is shown in Fig. 1a. Peripheral blood was collected and genomic DNA was isolated from currently available cases (II:2, III:1, III-5, and IV:1) and two unaffected family members (II:3 and III:3). The expansion of polyglutamine repeats in SCA1, SCA2, and SCA3 loci was excluded for all affected members of the pedigree. The family under investigation, as well as the 300 control individuals, were Russians of Slavonic origin.

### Whole-exome sequencing

WES was performed in two affected individuals (II-2 and IV-1) and in unaffected family member (II-3) at the NGS Core Facility of the Estonian Genome Center, University of Tartu, Estonia. Exome capture was performed with the TruSeq™ Exome Enrichment Kit (Illumina, USA), according to the manufacturer’s protocol. The captured libraries were sequenced with 100-bp paired-end reads on Illumina platform HiSeq2000. The in-house pipeline of the Estonian Genome Center, University of Tartu, was used for the alignment to the reference genome and variant calling, as described previously [5]. Because the SCA was an autosomal dominant hereditary disorder and only one parent was affected, we focused on heterozygous nonsynonymous, frameshift, and canonical splice-site variants that were absent from public datasets, including dbSNP137 [6], the 1000 Genomes Project [7], and the NHLBI Exome Sequencing Project (ESP) Exome Variant Server database [8]. Variants that were shared by two affected patients and were absent in a healthy family member were considered to be potentially related to the disease. Nonsynonymous amino acid variants were analyzed using SIFT [9], PolyPhen-2 [10], fathmm [11], MutationAssessor [12], and MutationTaster [13], to assess any potentially damaging effects. Protein-altering SNPs that were predicted to be damaging by at least two methods were considered as candidate causative variants. Relevant mutations in all SCA genes were then prioritized manually.

### Validation of selected variants

Candidate variant analysis was performed by Sanger sequencing of all available family members. PCR primers for *ITPR1* [NG_016144.1], *COL6A3* [NG_008676.1], *SCN9A* [NG_012798.1], *SYNE1* [NG_012855.1], and *COL9A1* [NG_011654.1] variants were designed using NCBI Primer-BLAST [14] (sequences and PCR conditions are available upon request). The purification of PCR products was performed using the Axygen Gel Extraction Kit. Sanger sequencing was performed in both forward and reverse directions on an ABI 3730 DNA Analyzer. Sequences were analyzed using the Sequence Scanner Software v.1.0 and AlignX® module for Vector NTI.

### Substitution characterization: mRNA secondary structure prediction and RSCU analysis

The analysis of the secondary structure of the ITPR1 mRNA and of associated Gibbs free energy (ΔG) values was performed essentially as described previously [15–17]. Gibbs free energy calculation was carried out using the mfold software [18] based on the nearest neighbor free energy model. The RNA structures with the lowest free energy for a given mRNA fragment (51 nt in length) were selected and the Gibbs free energy difference (ΔΔG = mutantΔG – WTΔG) was calculated (with the mutation of interest positioned in the middle of the mRNA fragment analyzed). It was shown previously that analysis of short mRNA fragments (~25–75 nt in length) was the most predictive [15–17].

Calculations of the differences in relative synonymous codon usage (RSCU) values [19] were carried out as described previously [15–17]. Briefly, the RSCU value for a mutant variant codon and that for the corresponding WT codon were calculated. ΔRSCU = RSCUmutant – RSCUwildtype represents a change in the RSCU values as a consequence of the mutation in the *ITPR1* gene and may be indicative of a change in translation rate around a particular codon.

## Results

### Clinical features of the family

**Fig. 2:**
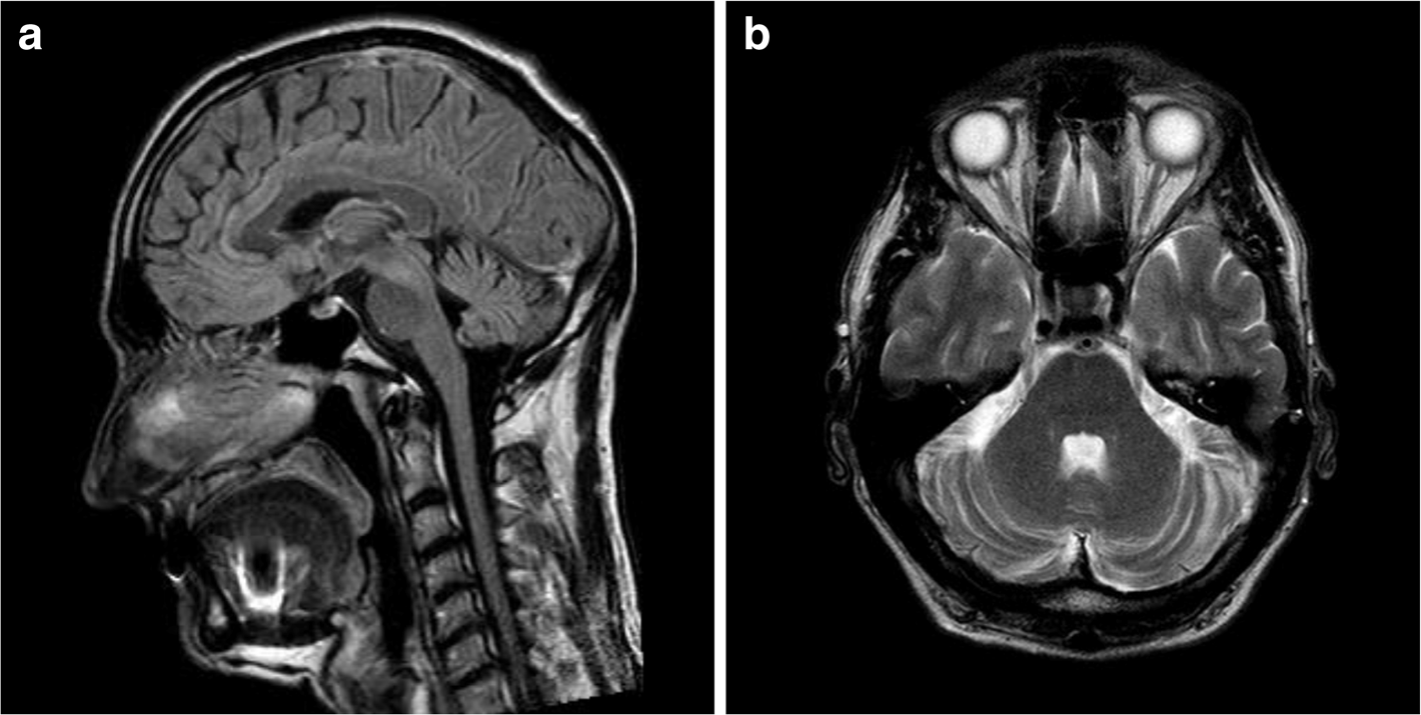
Cranial MRI of the proband (T2/FLAIR weighted sagittal **a**, and T2 weighted axial **b** images) showing mild cerebellar atrophy

The proband, patient A.P. (III-1), was 54 years old and was delivered normally after an uneventful pregnancy. The proband initially had some motor delay, began to walk at the age of 2, and his language and learning development was normal. He graduated from high school and college with satisfactory results. Despite being an agronomist by profession, he is currently employed as a caretaker. Clinically, for many years he suffered from unsteady walking, slowness of speech, and abnormal fine motor skills. The patient noted that some disease -related deterioration had taken place during his adolescence, including mild memory loss. Marked gait disturbance was observed at 15 years of age. On examination, he exhibited an unsteady wide-based gait, especially with his eyes closed, slightly slurred and slow speech, mild limb ataxia with intention tremor and dysmetria, mild truncal titubation, difficulties on tandem walking, and diffuse muscle hypotonia. The total scale for the assessment and rating of ataxia (SARA) score was 11. He had no nystagmus or paresis, and his deep tendon reflexes and sensitivity were normal. The patient has been under observation for 25 years: his clinical features were generally stable throughout the course of the disease, with a very mild increase in gait and speech difficulties over the last 8–10 years. Cerebral MRI scans demonstrated mild cerebellar atrophy (Fig. 2). Cognitive examination showed no clinically significant changes (Montreal cognitive assessment (MoCA) test score was 26).

According to the proband’s evidence, a similar disease affected his father (II-2) between 15 and 20 years of age. He had an unsteady gait and scanning speech throughout his life, but was able to live independently and died at the age of 81. The proband’s son (IV-1, 27 years of age) started walking independently only at 3 years of age and often fell during his childhood. Having graduated from high school and university with good results, he pursued studies in hi-tech; now he works successfully as an IT administrator. He also succeeded in learning a second language (English). The patient’s clinical features are very similar to those of his father, but his gait difficulties are more severe. The proband’s brother, who is 52 years old, also suffered from the same disorder and exhibited similar clinical features starting in adolescence.

### Next-generation sequencing (NGS) data

A total of 46507918, 50734956, and 40536854 reads were obtained for II:2, IV:1 and II:3, respectively. The average target coverage was 34.0× for II:2, 37.3× IV:1, and 29.0× for II:3.

### Identification of disease-causing mutations

WES identified 947 variants that were heterozygous in two affected patients and reference homozygous in one unaffected family member. These heterozygous variants were filtered through the databases SIFT, PolyPhen-2, fathmm, MutationAssessor, and MutationTaster; 12 matching variants were found that had a minor allele frequency (MAF) not exceeding 0.01. Among them, five variants were nonsynonymous and were predicted as damaging by at least two prediction methods.

### Validation of identified substitution

These five possibly pathogenic variants were analyzed via PCR and further Sanger sequencing in all available family members (Table 1). Only the p.Val1553Met (NM_001099952; NP_001093422) variant was identified in a heterozygous state in all four affected family members (II-2, III-1, III-5, and IV-1; see Fig. 1) and in a homozygous (Val1553Val, wild type) state in two healthy individuals (II-3 and III-3) (Fig. 1a, b). This mutation has been identified once previously in unrelated Australian family [20]. Next, we evaluated the frequency of the occurrence of this heterozygous variant in the control population, which consisted of 300 individuals. All samples analyzed were homozygous in this position (Val1553Val).

**Table 1:** Summary of variants revealed by whole-exome sequencing in the patients with SCA

Gene	SNP ID	Nucleotide/protein	Prediction by				
			SIFT	PolyPhen2	MutationTaster	MutationAssessor	fathmm
*ITPR1*	rs397514535	c.4657G>A p.Val1553Met	Tolerated	Probably damaging	Disease causing	Predicted non-functional (low)	Tolerated
*COL6A3*	rs146092501	c.4156G>A p.Glu1386Lys	Tolerated	Possibly damaging	Polymorphism	Predicted non-functional (low)	Damaging
*SCN9A*	rs41268673	c.1828C>A p.Pro610Thr	Tolerated	Benign	Disease causing	Predicted functional (medium)	Damaging
*SYNE1*	rs138787771	c.23315G>A p.Arg7772Gln	Tolerated	Benign	Disease causing	Predicted functional (medium)	Tolerated
*COL9A1*	rs77706858	c.1349A>G p.Glu450Gly	Tolerated	Probably damaging	Disease causing	Predicted non-functional (low)	Damaging

### mRNA secondary structure prediction and RSCU analysis

**Fig. 3:**
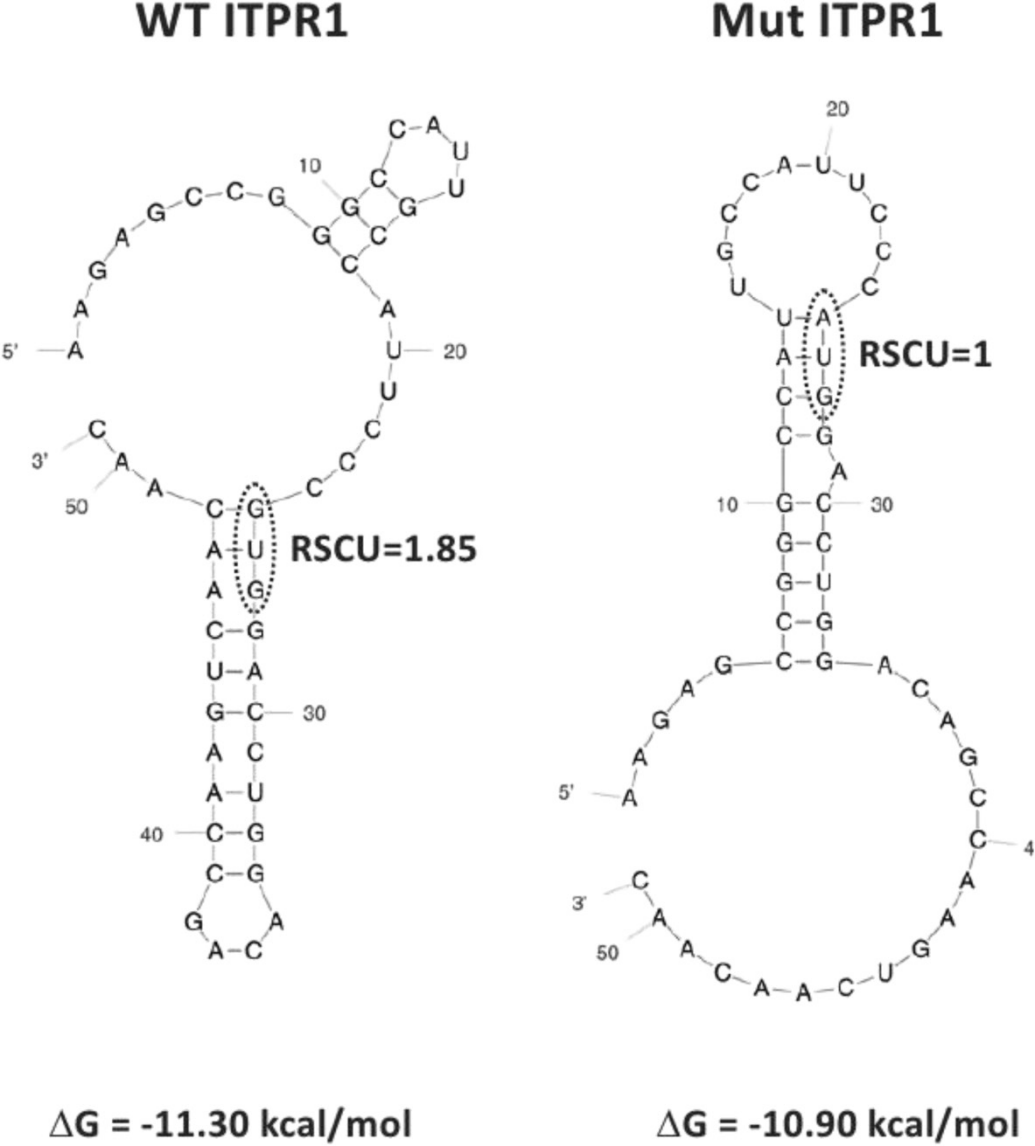
Comparative analysis of the ITPR1 mRNA structures (WT and G4684A (GUG > AUG) variant). WT and mutant 51 nt mRNA fragments, harboring the site of mutation in the middle were analyzed by mfold and shown. The site of mutation is circled by a dashed line. RSCU values for GUG and AUG codons are shown

We performed mRNA secondary structure prediction and RSCU analysis for the p.Val1553Met variant identified with positive family segregation. Our brief analysis of the G4684A *ITPR1* mutation, which changes a GUG(Val) codon into an initiator AUG(Met) codon, showed (Fig. 3) that this mutation led to substantial changes in Gibbs free energy (ΔG) (ΔΔG = 0.4), as well as in RSCU (ΔRSCU = –0.85). These changes may be indicative of a disease-causing mutation [16, 17].

## Discussion

As mentioned previously, SCAs represent a heterogeneous group of diseases from both the clinical and genetic points of view, as they include forms that are rare and difficult to diagnose. Therefore, a NGS analysis was needed to detect the underlying genetic variation. We performed an analysis of a family with a mild SCA clinical phenotype to identify the genetic variants that may contribute to the development of disease. One possibly pathogenic variant, p.Val1553Met, was identified in exon 37 of the *ITPR1* gene. We analyzed this mutation in all family members for whom DNA was available, and in the Russian population controls. The segregation of the identified substitution with the disease and its absence in the control Russian population as well as in the 1000 Genomes Project and the NHLBI ESP Exome Variant Server database were shown.

Prediction tests for this substitution indicated its pathogenic significance, as it was predicted as being probably damaging by PolyPhen-2 and as disease causing by MutationTaster.

Moreover, a recent genetic data analysis of patients with hereditary diseases revealed that not only the information contained at the amino acid sequence level of the target protein (mutant vs wild type) but also the information contained at the mRNA/nucleotide level may serve as a predictor of disease development and severity [16, 17]. In the case of blood disorders such as hemophilia A and B, which are caused by mutations in the *f8* and *f9* genes, respectively, it has been shown that statistically significant differences between severe and nonsevere disease-causing mutations can be observed at the mRNA level, i.e., severe disease-causing mutations tend to lead to substantial changes in the free energy of small mRNA fragments (harboring the site of mutation), and tend to lead to substantial changes in RSCU values. We evaluated the stability of the mRNA and the RCSU values for the possibly pathogenic variant identified, and found that it leads to a change in the structure and stability of the mRNA (ΔΔG = 0.4), as well as changes in RSCU (ΔRSCU = –0.85). Reported data indicate that such changes may affect the local translation rates, which can in turn perturb protein folding [21].

Thus, all listed data indicate that the G4684A substitution identified here, which results in a p.Val1553Met amino acid change, is disease causing and leads to the development of a form of SCA with a mild clinical phenotype in the family studied. This was supported by the fact that the involvement of *ITPR1* gene in the etiology of SCAs, SCA15 (former SCA16) and SCA29, was already shown [20, 22–24]. The same substitution was described previously in an Australian family, and the authors found another p.N602D substitution in the *ITPR1* gene in a Canadian family [20]. The neurological syndrome in the Canadian and Australian families was similar, with obvious anticipation and very slow clinical deterioration over many years. The lack of cognitive retardation in childhood and adolescence and the lack of clinically significant cognitive impairment in adulthood were important differences between our case and those occurring in the Australian and Canadian families.

To date, little is known about the role of the *ITPR1* gene in the pathogenesis of SCAs; however, we know that it encodes the type 1 inositol 1,4,5-trisphosphate (IP_3_) receptor, which is an intracellular IP_3_-gated calcium channel that modulates intracellular calcium signaling [25]. It releases calcium from the endoplasmic reticulum by binding to specific receptors that are coupled to calcium channels. These receptors are abundant in neuronal and non-neuronal tissues. Val1553 is located within the binding domain for carbonic anhydrase VIII (CA8). Its precise function is unknown, but it is assumed that CA8 competes with IP_3_ for binding to IP_3_R and inhibits the release of Ca from cells. It was supposed that the p.Val1553Met affects the cellular homeostasis of calcium, which is important for neuronal cells [26]. In addition, there is evidence of a direct physical interaction between ITPR1 and VCP proteins [27]. This protein is involved in the processes of neurodegeneration: mutations in VCP cause a pleiotropic degenerative disorder called multisystem proteinopathy, which can manifest clinically as classical amyotrophic lateral sclerosis, frontotemporal dementia, inclusion body myopathy, or a combination of these disorders [28, 29]. Furthermore, VCP interacts directly with ATXN3, which plays an important role in the pathogenesis of SCAs [17, 18]. Therefore, we can assume that changes in the amino acid structure of ITPR1 may indirectly affect the activity of VCP or ATXN3, which may contribute to the development of a neurodegenerative process in this type of ataxia.

## Conclusions

Thus, all existing data on the *ITPR1* genetic variation allows to presume that this gene plays an important role in the pathogenesis of SCAs, and, therefore, we suggest that the p.Val1553Met variant is a disease-causing mutation in the family studied.

## References

[1] Matilla-Duenas A, Corral-Juan M, Volpini V, Sanchez I (2012). The spinocerebellar ataxias: clinical aspects and molecular genetics. Adv Exp Med Biol..

[2] Harding AE (1982). The clinical features and classification of the late onset autosomal dominant cerebellar ataxias. A study of 11 families, including descendants of the ‘the Drew family of Walworth’. Brain.

[3] Hereditary Ataxias: Dominant [database on the Internet]. Neuromuscular Disease Center 2015. Available from: http://neuromuscular.wustl.edu/ataxia/domatax.html. Accessed: 28.01.2015.

[4] Rossi M, Perez-Lloret S, Doldan L, Cerquetti D, Balej J, Millar Vernetti P (2014). Autosomal dominant cerebellar ataxias: a systematic review of clinical features. Eur J Neurol.

[5] Nikopensius T, Annilo T, Jagomagi T, Gilissen C, Kals M, Krjutskov K (2013). Non-syndromic tooth agenesis associated with a nonsense mutation in ectodysplasin-A (EDA). J Dent Res.

[6] dbSNP Short Genetic Variations [database on the Internet]2014. Available from: http://www.ncbi.nlm.nih.gov/projects/SNP/index.html. Accessed: 19.09.2014.

[7] 1000 Genomes [database on the Internet]2014. Available from: http://www.1000genomes.org/home. Accessed: 6.11.2014.

[8] NHLBI Exome Sequencing Project (ESP) Exome Variant Server [database on the Internet]2014. Available from: http://evs.gs.washington.edu/EVS/. Accessed: 03.11.2014.

[9] SIFT [database on the Internet]. Available from: http://sift.jcvi.org/www/SIFT_chr_coords_submit.html. Accessed: Aug 2011.

[10] PolyPhen-2 prediction of functional effects of human nsSNPs [database on the Internet]. Available from: http://genetics.bwh.harvard.edu/pph2/. Accessed: 15.02.2012.

[11] fathmm, Functional Analysis through Hidden Markov Models [database on the Internet]. Available from: http://fathmm.biocompute.org.uk/. Accessed: May 2014.

[12] MutationAssessor [database on the Internet]. Available from: http://mutationassessor.org/. Accessed: 12.09.2009.

[13] Schwarz JM, Cooper DN, Schuelke M, Seelow D (2014). MutationTaster2: mutation prediction for the deep-sequencing age. Nat Methods.

[14] Primer-BLAST. http://www.ncbi.nlm.nih.gov/tools/primer-blast/. Accessed 2015.

[15] Edwards NC, Hing ZA, Perry A, Blaisdell A, Kopelman DB, Fathke R (2012). Characterization of coding synonymous and non-synonymous variants in ADAMTS13 using ex vivo and in silico approaches. PLoS One.

[16] Hamasaki-Katagiri N, Salari R, Simhadri VL, Tseng SC, Needlman E, Edwards NC (2012). Analysis of F9 point mutations and their correlation to severity of haemophilia B disease. Haemophilia.

[17] Hamasaki-Katagiri N, Salari R, Wu A, Qi Y, Schiller T, Filiberto AC (2013). A gene-specific method for predicting hemophilia-causing point mutations. J Mol Biol.

[18] Zuker M (2003). Mfold web server for nucleic acid folding and hybridization prediction. Nucleic Acids Res.

[19] Sharp PM, Tuohy TM, Mosurski KR (1986). Codon usage in yeast: cluster analysis clearly differentiates highly and lowly expressed genes. Nucleic Acids Res.

[20] Huang L, Chardon JW, Carter MT, Friend KL, Dudding TE, Schwartzentruber J (2012). Missense mutations in ITPR1 cause autosomal dominant congenital nonprogressive spinocerebellar ataxia. Orphanet J Rare Dis..

[21] Komar AA (2009). A pause for thought along the co-translational folding pathway. Trends Biochem Sci.

[22] Hara K, Fukushima T, Suzuki T, Shimohata T, Oyake M, Ishiguro H (2004). Japanese SCA families with an unusual phenotype linked to a locus overlapping with SCA15 locus. Neurology.

[23] Hara K, Shiga A, Nozaki H, Mitsui J, Takahashi Y, Ishiguro H (2008). Total deletion and a missense mutation of ITPR1 in Japanese SCA15 families. Neurology.

[24] Iwaki A, Kawano Y, Miura S, Shibata H, Matsuse D, Li W (2008). Heterozygous deletion of ITPR1, but not SUMF1, in spinocerebellar ataxia type 16. J Med Genet.

[25] Berridge MJ (1993). Inositol trisphosphate and calcium signalling. Nature.

[26] Hirota J, Ando H, Hamada K, Mikoshiba K (2003). Carbonic anhydrase-related protein is a novel binding protein for inositol 1,4,5-trisphosphate receptor type 1. Biochem J.

[27] Pearce MM, Wormer DB, Wilkens S, Wojcikiewicz RJ (2009). An endoplasmic reticulum (ER) membrane complex composed of SPFH1 and SPFH2 mediates the ER-associated degradation of inositol 1,4,5-trisphosphate receptors. J Biol Chem.

[28] Johnson JO, Mandrioli J, Benatar M, Abramzon Y, Van Deerlin VM, Trojanowski JQ (2010). Exome sequencing reveals VCP mutations as a cause of familial ALS. Neuron.

[29] Watts GD, Wymer J, Kovach MJ, Mehta SG, Mumm S, Darvish D (2004). Inclusion body myopathy associated with Paget disease of bone and frontotemporal dementia is caused by mutant valosin-containing protein. Nat Genet.

